# Pneumolysin: Pathogenesis and Therapeutic Target

**DOI:** 10.3389/fmicb.2020.01543

**Published:** 2020-07-02

**Authors:** Andrew T. Nishimoto, Jason W. Rosch, Elaine I. Tuomanen

**Affiliations:** Department of Infectious Disease, St. Jude Children’s Research Hospital, Memphis, TN, United States

**Keywords:** *Streptococcus pneumoniae*, pneumococcus, pneumolysin, cholesterol-dependent cytolysin, invasive pneumococcal disease, vaccine

## Abstract

*Streptococcus pneumoniae* is an opportunistic pathogen responsible for widespread illness and is a major global health issue for children, the elderly, and the immunocompromised population. Pneumolysin (PLY) is a cholesterol-dependent cytolysin (CDC) and key pneumococcal virulence factor involved in all phases of pneumococcal disease, including transmission, colonization, and infection. In this review we cover the biology and cytolytic function of PLY, its contribution to *S. pneumoniae* pathogenesis, and its known interactions and effects on the host with regard to tissue damage and immune response. Additionally, we review statins as a therapeutic option for CDC toxicity and PLY toxoid as a vaccine candidate in protein-based vaccines.

## Introduction

*Streptococcus pneumoniae* is a commensal organism responsible for a wide array of disease and is the source of considerable morbidity and mortality. Able to colonize the nasopharynx of adults and more frequently of children, *S. pneumoniae* manifests as infections of the ear, respiratory tract, and even severe, life-threatening sepsis and meningitis. The cholesterol-dependent cytolysin (CDC) pneumolysin (PLY), is critical at many steps of pneumococcal disease, working in concert with adhesins, invasins, and proteases to achieve pathogenesis (Cundell et al., 1995; Zhang et al., 2000; Radin et al., 2005; Orihuela et al., 2009). Consequently, PLY’s effects in the body are numerous and diverse – contributing to inflammation and bacterial penetration, causing direct damage to cells through pore-forming cytolytic activity, aiding bacterial escape through blocking complement activation, and being a key factor in host-to-host pneumococcal transmission (Berry et al., 1989b; Rayner et al., 1995; Berry and Paton, 2000; Alcantara et al., 2001; Rogers et al., 2003; Mitchell and Dalziel, 2014; Zafar et al., 2017). Recognizing the multifaceted role of PLY in host-pathogen interactions is therefore paramount to better understand pneumococcal infection and address it as a therapeutic target.

## Biology of Pneumolysin

Pneumolysin is a 471 amino acid CDC whose properties and characteristics have been closely studied since the early 20th century (Neill, 1926; Cohen et al., 1942; Walker et al., 1987). Structurally, PLY has four functional domains, which were initially attributed based on sequence similarity to the bacterial pore-forming toxin perfringolysin from *Clostridium perfringens* (Kelly and Jedrzejas, 2000a, b). Domains 1 and 3 are linked via domain 2 to the membrane-sensing C-terminal domain 4 (Lawrence et al., 2015; Marshall et al., 2015). Like other CDCs, PLY contains the highly conserved undecapeptide sequence known as the tryptophan-rich loop and a threonine-leucine amino acid pair involved in membrane-bound cholesterol recognition and binding (Nollmann et al., 2004; Farrand et al., 2010).

**FIGURE 1:**
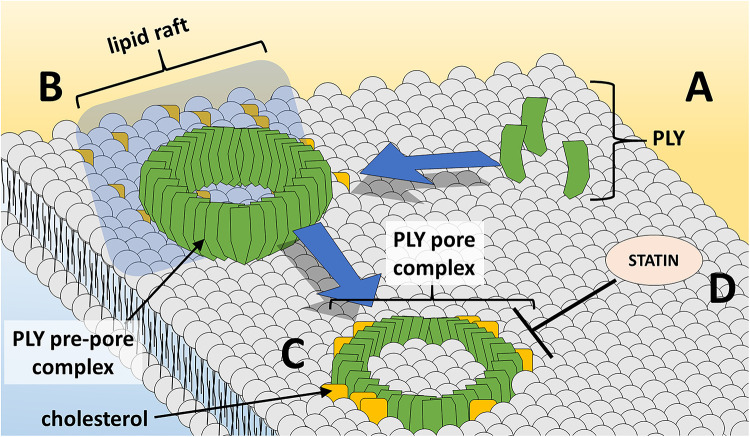
PLY pore formation at the host cell membrane. **(A)** PLY monomers gather at cholesterol-rich lipid rafts at the cell membrane and **(B)** assemble in the ring-shaped pre-pore complex. **(C)** Insertion of the PLY pore-forming complex into lipid bilayer results in loss of membrane integrity and cell damage/death. **(D)** Statin medications oppose PLY-induced pore-formation at the cell membrane.

During the course of pore-formation, PLY monomers bind to the targeted cell membrane and interact with other PLY molecules, packing side-by-side to form the pre-pore complex (Marshall et al., 2015) (Figure 1). After undergoing further conformational changes, the final ring-like pore of roughly 30–50 PLY subunits inserts into the membrane (Tilley et al., 2005). These pore and pre-pore formations at the cell membrane are initially shed in toxin-induced microvesicules as a mechanism of repair, with cytotoxic effects occurring via dysregulation of cell homeostasis through influx of calcium (Wolfmeier et al., 2016). Repeated insertion of numerous pore-forming PLY complexes can result in membrane destabilization, loss of ion homeostasis, and ultimately cell death. Intracellular calcium influx via formed pores at the cell membrane can trigger changes in host cell mitochondrial membrane ultrastructure, loss of mitochondrial membrane potential and release of mitochondrial apoptosis-inducing factor (Braun et al., 2007; Nerlich et al., 2018). In addition, PLY can induce double-strand breaks in cellular DNA resulting in loss of genomic integrity and further cytotoxicity (Rai et al., 2016). As will be discussed, PLY’s effects extend beyond cell damage and death, altering cells, cell components, and their respective functions to modulate and facilitate transmission, invasion, and colonization.

While it is popularly accepted that cholesterol is a PLY cellular receptor, this has not been definitively demonstrated with intact pneumococcal cells. Early studies observed PLY inhibition in the presence of cholesterol (Walker et al., 1987), possibly due to saturation of binding sites on PLY, indicating that membrane cholesterol may be the target receptor as suggested for other CDCs (Watson et al., 1972). Furthermore, cholesterol was shown to have specific 1:1 stoichiometric interactions with PLY and to be required for PLY hemolytic activity (Nollmann et al., 2004). Recent investigations studied lipid–lipid and lipid–protein interactions at different pore-forming stages of PLY upon exposure to cholesterol-containing liposomes. Gradual decreases in membrane order and increases in rotational diffusion of the lipids during pre-pore oligomerization stages but not during PLY oligomer binding and insertion into the membrane have been described (Faraj et al., 2020). Some evidence suggests that PLY-membrane cholesterol interactions may have more to do with toxin activation than cell binding (Soltani et al., 2007). Notably, PLY has been shown to interact with cellular glycolipid receptors at the surface of human red blood cells, supporting growing evidence that pore-forming toxins possess non-cholesterol receptors (Shewell et al., 2014).

Pneumolysin is not actively secreted by *S. pneumoniae* owing to a lack of an N-terminal secretion signal (Walker et al., 1987). It is the only member of the CDC class to behave in this manner, though interestingly this trait is universally conserved across pneumococci (Martner et al., 2008). While it was thought that PLY relied on the pneumococcal autolysin LytA and cellular autolysis for release (Berry et al., 1989a; Canvin et al., 1995), it was later revealed that the amount of extracellular PLY released by a *lytA-*mutant was comparable to the wildtype strain (Balachandran et al., 2001) and that PLY release can occur in early growth phases when the autolytic cascade is inactive (Benton et al., 1997) In the absence of autolysis, PLY localizes to the cell wall (Price and Camilli, 2009). Furthermore, domain 2 is required for PLY export to the cell wall compartment, and interestingly fusion of a secretion signal sequence to PLY did not result in detectable release of PLY protein (Price et al., 2012). While factors such as pyruvate oxidase enhance PLY release, possibly via intracellular H_2_O_2_-dependent mechanisms, *S. pneumoniae* may also modulate the release of PLY exported to the cell wall based on the composition of branched stem peptides and choline-binding proteins in the peptidoglycan cell wall network, and peptidoglycan remodeling by hydrolases may contribute to PLY release (Greene et al., 2015; Bryant et al., 2016).

## Pneumolysin in Host Tissue Injury

The pneumococcus is a highly successful pathogen that infects the middle ear, lungs, blood, heart, and brain. In the host, PLY works on multiple levels to facilitate invasion by contributing to direct cell damage and escape from the host’s immune system. It has long been known that PLY exhibits cytotoxic activity for virtually every cell type in the body. Lung endothelial cells as well as nasal and tracheobronchial epithelial cells are damaged as a result of pore-forming activity (Steinfort et al., 1989; Rubins et al., 1992; Rayner et al., 1995). Mutants lacking PLY fail to induce lung mucosal damage (Rubins et al., 1995). Acute lung injury has also been attributed to PLY’s effects on specific host factors, such as the increased release of platelet activating factor, which contributes to proinflammatory actions, increased vascular permeability, and vasoconstriction (Witzenrath et al., 2007). Pneumolysin can activate platelets *in vitro* via calcium influx due to sub-lytic pore formation, which may similarly potentiate lung inflammation (Nel et al., 2016).

In addition to the lung, invasive pneumococcal disease can also lead to cardiac pathologies. For example, intraperitoneal injections into mice with *S. pneumoniae* led to microlesions in the ventricular myocardium, indicating bacterial translocation into the heart (Brown et al., 2014). Fluorescence microscopy showed that PLY localized to these microlesions and induced cardiomyocyte injury (Alhamdi et al., 2015). Furthermore, even sub-lytic PLY concentrations induced cardiomyocyte dysfunction via calcium influx and a resulting reduction in contractility.

Pneumolysin plays a key role in pathogenesis of pneumococcal meningitis by facilitating bacterial crossing of the blood-brain barrier. *In vitro* models using human and bovine brain microvascular endothelial cells indicated that PLY induces loss of tight junctions, and this endothelial cell damage may contribute to bacterial translocation (Ring et al., 1998; Zysk et al., 2001). Additionally, PLY-induced remodeling of brain tissue through astrocyte reorganization, in combination with interstitial fluid retention, may promote bacterial invasion (Hupp et al., 2012). Pneumolysin released in the CSF directly damages the ependymal cells lining the ventricles of the brain, resulting in loss of cilia and reduction in ciliary beat frequency indicative of decreased cell viability and flow of cerebrospinal fluid (Mohammed et al., 1999; Hirst et al., 2000a). Subsequently, additional studies have demonstrated that PLY-deficient strains possessed decreased virulence in murine meningitis and, in rabbit models, decreased hippocampal damage via neuronal apoptosis (Braun et al., 2002; Wellmer et al., 2002).

Pneumolysin additionally affects pathogenesis in acute otitis media. Using chinchilla otitis models, the PLY deletion mutant yielded a reduced recovery of bacteria from middle ear fluid and reduced biofilm formation (Keller et al., 2016). Pneumolysin deletion resulted in reduced pathological changes to the round window membrane and less recovered bacteria from middle ear effusions, supporting PLY’s role in virulence during middle ear infections (Schachern et al., 2013).

Pneumolysin demonstrates not only cytolytic activity, but direct interference with host responses. Pneumolysin shows complement-activating and complement consumption properties that divert opsonization and subsequent phagocytosis of intact pneumococcal cells (Paton et al., 1984; Mitchell et al., 1991; Rubins et al., 1995). An early *in vitro* study showed that PLY inhibited polymorphonuclear leukocytes by interfering with opsonization, reducing respiratory burst response and decreasing leukocyte migratory ability (Paton and Ferrante, 1983). Within the lungs, PLY acts to help *S. pneumoniae* escape detection via creating a refuge inside dendritic cells. At low doses, PLY binds to the mannose receptor C type 1 (MRC-1) on dendritic cells and, in addition to inhibiting proinflammatory cytokine release, causes dendritic cells to internalize *S. pneumoniae* (Subramanian et al., 2019). Pneumolysin’s interaction with MRC-1 also inhibits pneumococcal-infected vacuoles from fusing with lysosomes, promoting intracellular pneumococcal survival (Subramanian et al., 2019).

It is especially important to note that many murine studies may underestimate the actual contributions of PLY to pathogenesis. Compared to human serum, pooled mouse serum had a greater ability (62-fold vs. >3000-fold change in EC_50_) to inhibit PLY hemolytic activity (Wade et al., 2014). Closer inspection revealed that cholesterol carried by mouse ApoB-100 lipoprotein, but not human ApoB-100, possessed potent PLY inhibitory activity, causing premature pore formation and subsequent inactivation of PLY’s lytic activities. Consequently, PLY studies involving mice must be closely examined, as these findings imply that PLY activity in mice may underestimate PLY’s role in human disease and that mouse models expressing human ApoB-100 may represent a better translation to human infections.

## Pneumolysin in Transmission and Colonization

Host-to-host transmission is an important component in the development of pneumococcal infection in which PLY also plays a role. In a study evaluating *S pneumoniae* transmission between mouse pups, recombinant PLY administered intranasally in doses of 100 ng/pup or greater caused increased levels of bacterial shedding in nasal secretions (Zafar et al., 2017). This increased shedding appeared to be related to acute inflammation since wildtype recombinant PLY resulted in increased proinflammatory cytokine IL-1β, while a mutant PLY_*W433F*_, defective in pore-formation, did not show increased inflammation or bacterial shedding. Furthermore, only strains expressing native PLY, and not the PLY-deficient or mutant PLY_*W433F*_ strains, successfully transmitted (Zafar et al., 2017).

*S. pneumoniae* is known to form biofilms when colonizing the nasopharyngeal passages and middle ear (Hoa et al., 2009). Biofilm formation plays a significant role in fomite-mediated pneumococcal transmission, allowing *S. pneumoniae* to survive longer in the environment (Marks et al., 2014). Also, pneumococcal biofilms appear more suited for enhancing colonization and transmission, at the cost of attenuated virulence (Gilley and Orihuela, 2014). Pneumolysin-deficient mutants show significantly reduced biofilm formation compared to the wildtype, even though PLY’s contribution to biofilm development is not dependent on hemolytic ability (Shak et al., 2013; Keller et al., 2016).

## Pneumolysin as a Mediator of Cell Death and Inflammation

Due to its pore-forming cytolytic activity, PLY is capable of damaging and killing multiple cell types not just by necrosis but also by triggering programmed cell death pathways (Table 1). This occurs in lung and myocardial tissue during pneumococcal invasive disease and applies to other pneumococcal infections as well (Steinfort et al., 1989; Rubins et al., 1992; Alhamdi et al., 2015; Rashwan et al., 2018). For example, PLY has been shown to induce severe damage to cochlear cells resulting in hearing loss and deafness (Winter et al., 1997). In rat cochlear hair cells, PLY showed preferential killing of inner vs. outer cochlear hair cells via mitochondria-mediated apoptosis due to intracellular increases in calcium (Beurg et al., 2005). Induction of apoptosis is a major mechanism of brain injury in meningitis (Braun et al., 1999, 2001, 2002; Mitchell et al., 2004). Waves of apoptosis are seen in cortical and hippocampal neurons and in microglia in mouse models (Braun et al., 2007). Pneumolysin colocalizes with apoptotic neurons, and damage is significantly decreased during infection by PLY-/- strains. Thus, PLY is a major determinant of the poor outcome and permanent sequelae of meningitis.

**TABLE 1:** Classifications of PLY-induced cell death pathways.

**Cell death**	**Affected tissue**	**References**
Apoptosis	Cochlear hair cells	Beurg et al., 2005
	Cortical neurons	Braun et al., 2002, 2007
	Dendritic cells	Littmann et al., 2009
Necroptosis	Respiratory epithelium	Gonzalez-Juarbe et al., 2017
	Alveolar macrophages	Riegler et al., 2019
Direct cytotoxicity	Lung endothelium	Rubins et al., 1992
	Nasal/tracheobronchial epithelium	Steinfort et al., 1989;Rayner et al., 1995
	Polymorphonuclear leukocytes	Johnson et al., 1981
	Platelets	Johnson et al., 1981
	Cardiomyocytes	Alhamdi et al., 2015
	Brain microvascular endothelium	Zysk et al., 2001
	Ependymal cells	Mohammed et al., 1999; Hirst et al., 2000b

In addition to the necrotic cell death accomplished directly by PLY, the pneumococcal CDC can also induce programmed necroptosis in respiratory epithelial cells and alveolar macrophages (Gonzalez-Juarbe et al., 2017). By inhibiting regulatory kinases (RIP1, RIP3, and MLKL) in the necroptosis pathway, mouse macrophages or human macrophage-like THP-1 cells were partially protected from PLY-induced cell death (Gonzalez-Juarbe et al., 2015). Necroptosis was detected in infiltrating macrophages during *S. pneumoniae* invasion of the myocardium and was believed to contribute to adverse remodeling in the heart (Gilley et al., 2016). A PLY-dependent increase in nasopharyngeal epithelial cell necroptosis was observed in asymptomatically colonized mice and indicated that this may benefit bacterial clearance at early stages of mucosal colonization (Riegler et al., 2019).

Pneumolysin itself is also a critical mediator of inflammation. An *in vivo* study in rats showed that PLY injection alone replicated the inflammation and histological findings consistent with pneumococcal pneumonia and found that PLY, independent of hemolytic activity, induced considerable pneumonitis *in vivo* (Feldman et al., 1991). *In vitro*, *S. pneumoniae* increases expression of proinflammatory cytokines like IL-1β and TNF-α in human epithelial cells (Yoo et al., 2010). A calcium-dependent increase in the proinflammatory mediators leukotriene B_4_ and prostaglandin E_2_ has also been seen *in vitro* when examining human neutrophils treated with recombinant PLY, further supporting PLY’s proinflammatory role during invasive disease (Cockeran et al., 2001).

Multiple mechanisms are responsible for the inflammation in response to PLY. Pneumolysin’s ability to activate the classical complement pathway likely contributes to generalized inflammation through the host’s release of C3a and C5a anaphylatoxins (Paton et al., 1984). Several studies have also identified toll-like receptor (TLR) 4 for its role in recognizing PLY and subsequent inflammatory response regulation in murine and human cells (Malley et al., 2003; Bernatoniene et al., 2008; Dessing et al., 2009). Additionally, mice defective for TLR4 signaling were found to be hypersusceptible to pneumococcal disease and more susceptible to nasopharyngeal colonization with *S. pneumoniae*, suggesting that TLR4 is key to orchestrating proper response to pneumococcal disease.

PLY is necessary for IFN-γ and IL-17A induction in *in vivo* murine pneumonia models, suggesting that PLY is required for release of some inflammatory cytokines while synergistic with TLR agonists to enhance secretion of others. Of particular significance was the finding that PLY activates the NLRP3 inflammasome in order to induce the processing and secretion of IL-1β from dendritic cells, and interestingly, the PLY-induced cytokine release from mouse dendritic cells was found to be independent of TLR4 (Mcneela et al., 2010). More recently, PLY has been suggested to incite inflammatory cytokine release in neutrophils and THP-1 monocyte-derived macrophages, but actually inhibit release of TNF-α, IL-1β, and IL-12 in human-derived dendritic cells (Subramanian et al., 2019). Infection of dendritic cells showed a STAT1- and NF-κB-dependent inhibition of proinflammatory cytokine release compared to infection with a PLY-deficient mutant. The observed interaction of PLY with the phagocytic receptor, MRC-1, which is highly expressed in dendritic cells compared to neutrophils or GM-CSF derived macrophages, suggests that this dose-dependent inhibition of inflammatory cytokines aids in allowing *S. pneumoniae* to establish intracellular residency within the dendritic cells (Subramanian et al., 2019). Moreover, these mechanisms may help identify PLY’s contribution to inflammation in the nasopharynx, as MRC-1-expressing macrophages have been observed to be present in low-density pneumococcal carriage (Neill et al., 2014).

## Pneumolysin as a Therapeutic Target

To date, there has been limited study of non-antibiotic strategies for the prevention or improvement of outcome of pneumococcal infection. Statins, the popularly prescribed cholesterol-lowering medication class, inhibit 3-hydroxy-3-methylglutaryl CoA reductase – the rate-limiting step in cholesterol biosynthesis. Furthermore, statins possess beneficial immunomodulatory and anti-inflammatory properties in infection and cardiovascular disease, and some evidence supports a beneficial effect of statins in community-acquired pneumonia outcomes (Ray and Cannon, 2005; Terblanche et al., 2007; Chalmers et al., 2008). One study demonstrated efficacy in using statins to inhibit cell-damaging PLY activity (Figure 1) to improve outcomes in sickle-cell mice (Rosch et al., 2010). Patients with sickle-cell disease experience a 600-fold increase in lethality due to pneumococcus and thus, sickle-cell mice are an excellent animal model to test interventions. Pretreatment of sickle-cell mice with simvastatin resulted in decreased mortality, pneumococcal burden in the lungs and bloodstream, and severity of lung pathology.

Based on this study, statins seemed to have a two-pronged effect on pneumococcal infection. First, statin’s anti-inflammatory activity reduced the expression of platelet activating factor receptor (PAFr) in mouse lungs and vascular endothelium, thus reducing PAFr-mediated bacterial endocytosis and invasion. Second, statins directly reduced PLN cytotoxicity *in vivo* and *in vitro* at physiologic concentrations. Protection extended to tetanolysin and streptolysin, suggesting that therapeutic statin levels may be sufficient to impact CDC toxicity in general (Rosch et al., 2010). This was further shown not only for simvastatin and PLN but also for pravstatin protection of airway epithelial cells treated with alpha-hemolysin Statt et al., 2015). Evidence for a commercially available drug class to alter PLY function has far-reaching implications for immediately improving outcomes of pneumococcal infection. Moreover, if drug-mediated inhibition of PLY extends its roles in transmission and colonization, these agents may prevent spread of pneumococcal disease. Future studies are needed to investigate the mechanisms by which statins may affect PLY and prevent fulminant pneumococcal disease and whether their beneficial effects persist in combination with current antibiotic therapies.

It is also important to note the existence of other natural compounds that have been studied as inhibitors of PLY. β-sitosterol, for instance, was shown to bind with high affinity to PLY, inhibit PLY-induced hemolysis, protect human alveolar epithelial cells from injury, and prevent lethal pneumococcal infection (Li et al., 2015). Investigation of other natural compounds have identified chemical moieties important to blocking of PLY hemolytic activity and perhaps to the design of future inhibitors of PLY (Li et al., 2017). Similar studies have also reported prevention and attenuation of pneumococcal infection using compounds that prohibit PLY oligomerization (Li et al., 2020; Lv et al., 2020; Xu et al., 2020). These compounds potentially show promise as tools for developing small molecule adjuvant therapies in the prevention or treatment pneumococcal infection and studying PLY interactions.

The presence of anti-PLY antibodies has shown protective effects in delaying time to first pneumococcal carriage in newborns (Holmlund et al., 2006; Francis et al., 2009). Conversely, there is supportive evidence that patients with low serum antibody to PLY may be at higher risk for developing pneumococcal pneumonia (Huo et al., 2004). Mice administered anti-PLY antibodies showed significantly lower nasopharyngeal colonization compared to mice given nonspecific control antibodies (Kaur et al., 2014). It is therefore not surprising that PLY is one among several pneumococcal antigens studied as a potential vaccine candidate (Rapola et al., 2000; Holmlund et al., 2006; Francis et al., 2009). Pneumococcal protein based vaccines are particularly attractive in that the antigens are independent of capsular serotype and therefore may provide additional coverage compared to current serotype-based pneumococcal vaccines or may enhance protection when conjugated to capsular polysaccharide (Paton et al., 1991; Feldman and Anderson, 2014). Even so, PLY may present some challenges to its effective development as a vaccine target. For example, PLY is released from cells into the supernatant and thus is not present on the pneumococcal cell surface where an antibody could promote opsonization.

Fortunately, PLY shows relatively limited variation between strains and serotypes (Han and Zhang, 2019). Furthermore, PLY cytolytic effects can easily be attenuated with single amino acid substitutions (Boulnois et al., 1991). Specifically, a PLY L460D amino acid substitution disrupting the conserved CDC cholesterol-recognition motif abolishes any detectable cytotoxic effects (Farrand et al., 2010; Chen et al., 2015). This, in combination with the importance of PLY in immune cell recognition, makes PLY toxoid a key component in many protein-based vaccine trials (Malley et al., 2003; Witzenrath et al., 2011). Pneumolysin and attenuated PLY toxoid have since been extensively studied in animals for protective effects as an immunogenic agent (Paton et al., 1983; Alexander et al., 1994; Musher et al., 2001; Ogunniyi et al., 2001; Garcia-Suarez Mdel et al., 2004; Sanders et al., 2010; Denoel et al., 2011; Lu et al., 2014; Hermand et al., 2017). Many pre-clinical studies have additionally investigated protein-based vaccines using PLY toxoid combined with additional surface antigens. For example, a vaccine utilizing fusions of PLY toxoid and the major pneumococcal virulence factor choline-binding protein A was found to have broadly protective activities against pneumococcal infection in mouse nasopharyngeal carriage and infection models of sepsis, meningitis, otitis media, and pneumonia (Mann et al., 2014). Immunization with PLY-peptide fusions results in expanded PLY epitope recognition, indicating such fusions may engender enhanced protective capacity by facilitating recognition of protective epitopes that typically do not elicit a robust antibody response (Mann et al., 2014). While antibody responses against PLY are clearly one important component in protective immunity against *S. pneumoniae*, protective immunity requires responses against multiple protein antigens underscoring the important of multicomponent vaccines (Wilson et al., 2017).

Human trials using PLY-based vaccines have also been successful in demonstrating protection and immunogenicity (Bologa et al., 2012; Seiberling et al., 2012; Berglund et al., 2014). A detoxified PLY-derivative given to healthy adult humans demonstrated increased IgG titers against the PLY toxoid and increased toxin-neutralizing antibody activity (Kamtchoua et al., 2013). However, a pneumococcal vaccine containing PLY toxoid has yet to show protection in humans. Phase II trials showed no additional protection against infant pneumococcal nasopharyngeal carriage, acute otitis media or pneumonia by adding PLY toxoid in combinations with other antigens (Odutola et al., 2017; Hammitt et al., 2019). This suggests that PLY toxicity may not be a major driver of mucosal disease but the question remains open, as it is generally accepted to be an important component of future protein-based vaccines. As vaccine development continues, the efficacy, safety, and immunogenicity of PLY-based vaccines should be reassessed not only for effectiveness against disease prevention and carriage but also for changes in immune response mediated by PLY.

## Conclusion

The CDC PLY has multiple interactions with the host leading to extensive spread of disease, intense inflammation and abundant cell damage. Pneumolysin appears to incite tissue damage to increase bacterial penetration and transmissibility while simultaneously disarming elements of host defense. These effects of PLY promoting virulence are balanced against increased bacterial clearance and enhanced immune responses induced via cell damage and inflammation; thus, the timing, magnitude, and localization of PLY release can have a major impact on invasion and host response. Attenuation of tissue damage by statins that interfere with cholesterol in membranes may be a feasible therapeutic strategy for CDCs. Current research into PLY as a vaccine target shows promise especially when addressing infection from strains not currently covered in commercially available pneumococcal vaccines or non-typeable *S. pneumoniae*.

## Author Contributions

JR and ET conceived the manuscript and provided the groundwork of relevant content. AN drafted the manuscript. All authors reviewed and edited the manuscript.

## Conflict of Interest

The authors declare that the research was conducted in the absence of any commercial or financial relationships that could be construed as a potential conflict of interest.
